# Case report: Cavernous hemangioma in the right frontoparietal junction

**DOI:** 10.3389/fsurg.2022.972641

**Published:** 2022-08-30

**Authors:** Xuemin Cao, Xiaoshuai Chen, Yi Wang, Shangang Feng, Zengwu Wang

**Affiliations:** ^1^ Department of Neurosurgery, Weifang People's Hospital Affiliated to Weifang Medical University, School of Clinical Medicine, Weifang Medical University, Weifang, China; ^2^ Department of Neurosurgery, Weifang People's Hospital Affiliated to Weifang Medical University, Weifang, China; ^†^ These authors share first authorship

**Keywords:** cavernous, hemangioma, intraosseous hemangioma, skull, bone neoplasms

## Abstract

**Background:**

Primary intraosseous cavernous hemangioma is a benign tumor with slow growth and is rarely seen in clinics. The clinical manifestations of most patients are progressive enlargement of the head mass.

**Case presentation:**

We report a 30-year-old female patient with cavernous hemangioma at the frontoparietal junction. Upon admission, the right frontal lobe mass was progressively enlarged for 3 years and underwent lesion resection and stage I skull reconstruction. The postoperative outcome was good, with no recurrence at 1-year follow-up.

**Conclusion:**

Primary intraosseous cavernous hemangioma is a relatively rare clinical tumor, the pathogenesis of which is still unclear, and most of them have no specific clinical manifestations. Characteristic imaging findings are highly suspicious of this disease, but the definitive diagnosis still depends on histopathological examination. Currently, total surgical resection of the tumor is a relatively effective and preferred treatment.

## Introduction

Primary intraosseous cavernous hemangiomas (PICHs) are benign, slow-growing tumors that are clinically rare, accounting for approximately 0.2% of all bone tumors, and can occur in different parts, often in the frontal bone (1). PICHs are mostly isolated lesions that are mostly confined to the same skull (2). Multiple occurrences are rare, and the same lesion spanning the bony suture is even rarer. Most patients present clinically with a painless progressively enlarging mass in the head with no abnormalities on physical examination. For rare clinical symptoms, most of the clinical manifestations are related to the destruction of bone and compression of intracranial functional areas by the enlarged tumor. Relevant literatures are mostly in the form of case reports. In this paper, we report a case of cavernous hemangioma in the frontoparietal junction and discuss its clinical features, imaging manifestations, and treatment modalities. The retrospective report is as follows.

## Case description

**Figure 1:**
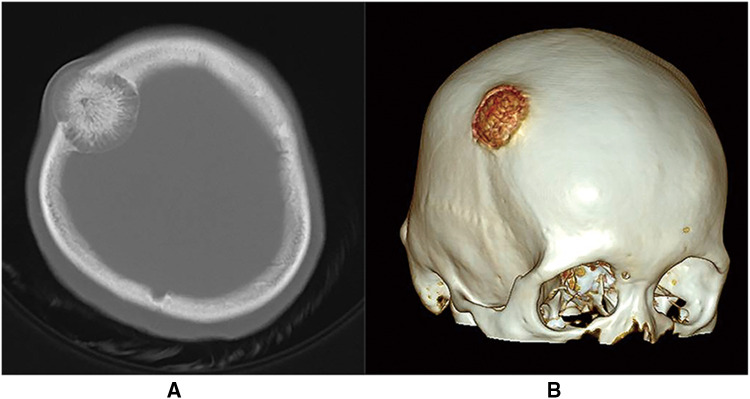
(**A**) Cranial CT scan (bone window) showing a right frontoparietal junctional area mass with distending growth, accompanied by osteolytic changes, showing the phenomenon of “daylight radiation”. (**B**) Cranial CT 3D reconstruction.

**Figure 2:**
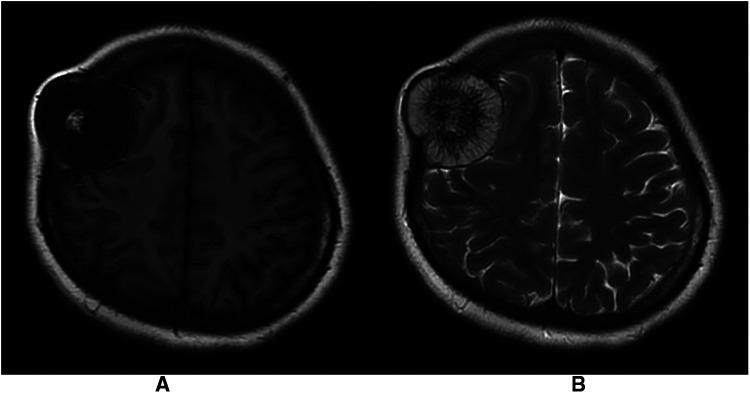
(**A**) Mixed long T1 signal shadow with irregular short T1 signal shadow in T1-weighted images. (**B**) Mixed long T2 signal shadow with radiolucent short T2 signal shadow in T1-weighted images.

**Figure 3:**
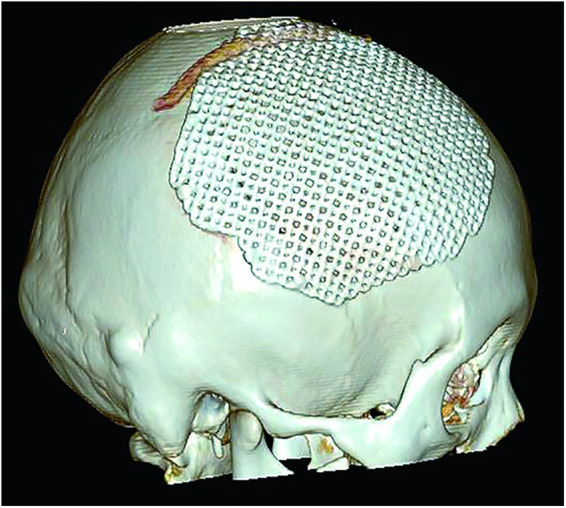
Postoperative cranial reconstruction.

**Figure 4:**
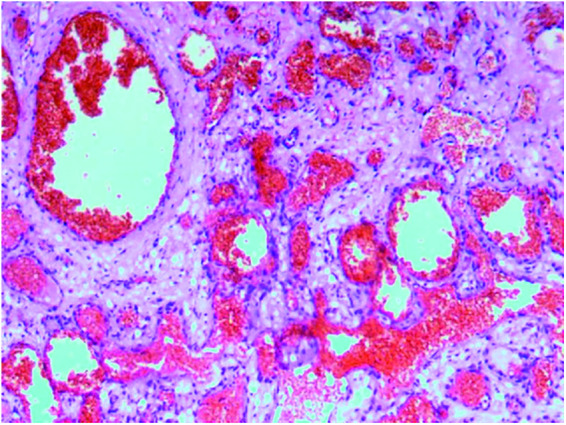
Postoperative pathology report, suggesting cranial cavernous hemangioma. Histopathologic view of the resected tumor: hematoxylin and eosin (H&E) staining.

The patient was a 30-year-old female who had a progressive enlargement of the right frontal mass for 3 years. Physical examination at admission showed a right frontal mass, which was hard, had no tenderness, and had poor range of motion. The patient was conscious. The bilateral pupils were equally large and rounded, and the reflex to light was sensitive. The neck was soft. The limbs could move as instructed, and the muscle strength and tension of the limbs were normal. The limbs appeared to be normal. Physiological reflexes were normal, and no pathological signs were elicited. CT scan manifestation showed no abnormal density shadow in all layers of brain parenchyma, round swelling bone changes in right frontoparietal junction, thinning of bone cortex, radiolucent internal bone, uneven density, and adjacent brain tissue compression. Bone changes in the right frontoparietal junction, benign bone tumor, or meningioma cranial changes were also observed (Figure 1). Cranial MRI showed axial T2-weighted images, axial-weighted images, and sagittal T1-weighted images; and the FLAIR sequence showed no significant abnormal signal shadows in the brain parenchyma of bilateral cerebral hemispheres. The midline structure was slightly left deviated. In the right frontoparietal bone, a round-like mixed T1 and long T2 signal shadows were seen, and irregular or radial crown short T1 and short T2 signal shadows were seen within it, with restricted diffusion of the edges, local thinning of the bone cortex, protrusion to the inside and outside, and displacement of the adjacent brain tissue by pressure. The right frontoparietal bone was occupied, mostly benign, and cranial hemangioma was considered (Figure 2). The patient's serum biochemical index results, including blood routine tests, C-reactive protein, tumor markers, and coagulation function, were all normal. Under general anesthesia with tracheal intubation, the right frontal horseshoe-shaped surgical incision was marked, and the skull was drilled about 0.5 cm from the edge of the occupancy. The bone flap was milled with a milling cutter to form a bone window approximately 6 cm × 6 cm in size, and the occupancy was seen to be adhering to the dura mater inwardly with a hard texture and general blood supply. The tumor was removed completely, the titanium plate was fixed to the bone edge with 10 titanium nails, and the tracheal tube was removed and returned to the ward after the patient awoke. Postoperative symptomatic treatments such as dehydration, nerve stimulation, nutrition, and fluid replacement were given. On the first day after surgery, the cranial CT was reviewed, and the surgical result was good with satisfactory reconstruction (Figure 3). The postoperative pathology was confirmed as cranial cavernous hemangioma (Figure 4), and the patient was discharged 11 days after surgery in good condition.

## Discussion

PICH is a rare benign tumor, accounting for approximately 0.2% of all bone tumors. The incidence occurs in 40- to 50-year-olds. It can occur in both men and women, with a male-to-female ratio of 3:1 to 2:1 (1, 2). PICH is mostly an isolated lesion (3) that is mostly confined to the same skull. However, in our patient, PICH is located between the frontal and parietal bones, which is rare.

Its pathogenesis is still unclear at this point, but some scholars (4) suggested that PICH may be heredity and caused by *KRIT1*/*CCM1* gene frameshift mutation. Others (5) suggested that hemangioma originates from undifferentiated mesenchymal tissue and trauma may induce differentiation and proliferation of undifferentiated mesenchymal tissue, which could be one of the pathogenesis of cavernous hemangioma. In this case, there was no history of trauma and family history of the disease. In conclusion, the pathogenesis of PICH is unclear and needs to be confirmed by further research.

PICHs mostly have no obvious clinical symptoms, and most of them are painless progressive enlarging masses. Sixteen patients with PICH reported by Chengjun Wang et al. (6) mainly showed clinical manifestations of swelling, local pain, dizziness or headache, and even tongue extension and deviation. Some studies (7) showed that facial paralysis, hemifacial spasm, and even hearing and vestibular dysfunction can occur when hemangioma involves the temporal bone. The clinical manifestations of patients are mostly related to the tumor's growth site. The patient in this case did not present with unusual clinical manifestations.

CT is the most commonly used imaging examination. PICH is characterized by mostly expansive growth, showing an expansion of the plate barrier, mostly osteolytic destruction, thin borders, integrity of the internal and external plates of the skull. There are radial bone septa and radial bone needles perpendicular to the inner plate of the skull. It shows typical osteolytic “honeycomb,” “soap bubble,” or “sunburst” changes (1, 8–10). MRI scan showed that tumor signal is generally heterogeneous, while signal intensity varies, and signal characteristics are heavily influenced by the amount of slow-flowing venous blood and the ratio of red bone marrow to transformed bone marrow. Smaller lesions or lesions with more fatty components tend to show higher signal intensity in T1-weighted images, whereas larger lesions tend to show lower signal intensity. In the T2-weighted sequence, signal intensity is increased in areas with slow venous blood flow or with blood accumulation (10, 11). This is the same as the case we reported. However, it has also been reported (3) that a patient with PICH showed a dural tail sign in MR, which was mainly due to the noninvasive superficial growth of the tumor. Although there are distinguishing imaging features such as “sunburst,” “honeycomb,” or “soap bubble,” these features are not specific. Therefore, it is necessary to compare eosinophilic granuloma, meningioma, fibrous dysplasia, meningioma, fibrous anomalous hyperplasia, cystic fibrositis, osteoma, osteosarcoma, multiple myeloma, Langerhans cell histiocytosis, and epidermoid cysts (10, 12, 13). Central nervous system metastasis of prostate cancer can also result in the manifestation of primary bone tumors (14), but the patient did not have a history of prostate cancer, so we ruled out this diagnosis. In this case, we also included PICH in the preoperative diagnosis based on CT scan and MRI hints for bone tumor and meningioma, and the postoperative pathological findings were PICH. Therefore, although these imaging examinations are not specific, they enable us to consider making a preliminary diagnosis more comprehensively, thus reducing the risk of misdiagnosis and mistreatment.

For patients with PICH, surgical complete resection of the entire tumor is often an effective and preferred treatment option (7). One study showed that the removal of more than 0.5 cm of bone at the edge of the tumor can be successfully performed without tumor recurrence (9). For fast-growing tumors and high risk of bleeding, preoperative embolization can be chosen to reduce intraoperative bleeding (15), whereas, for larger PICH, brain digital subtraction angiography (DSA) examination can help to make preoperative surgical planning (6). For some patients with inoperable lesions, radiotherapy can be used as a treatment; however, radiotherapy alone can only inhibit tumor growth but not eradicate lesions, so there is a risk of carcinogenesis (7). Asymptomatic patients can also receive regular medical observation. In the case of PICH reported in this paper, according to the preoperative imaging examination, the outer surface of the tumor was smaller, while the plate barrier and the inner surface were larger, and further development might cause some clinical symptoms due to the compression of brain tissue. At the same time, considering the easy bleeding of hemangioma and the safety of operation, we chose to remove the focus completely and used titanium mesh for stage I skull reconstruction and achieved good results. The patient recovered well after the operation without complications. There was no recurrence of the tumor during the 1-year follow-up.

## Conclusion

In summary, PICH is a rare clinical tumor, the pathogenesis of which is still unclear, and there may be specific clinical manifestations depending on the location of tumor growth, but most of the patients come to the hospital with progressive enlarging masses. Although characteristic imaging findings are highly suspicious of the disease, definitive diagnosis still depends on histopathological examination. At present, surgical total excision of the tumor is the more effective treatment, but for those tumors with a special location or that were unresectable, radiotherapy can be considered.

## Data Availability

The original contributions presented in the study are included in the article/Supplementary Material, further inquiries can be directed to the corresponding author/s.
